# The epigenetically downregulated factor CYGB suppresses breast cancer through inhibition of glucose metabolism

**DOI:** 10.1186/s13046-018-0979-9

**Published:** 2018-12-13

**Authors:** Yixiao Feng, Mingjun Wu, Shuman Li, Xiaoqian He, Jun Tang, Weiyan Peng, Beilei Zeng, Chuxia Deng, Guosheng Ren, Tingxiu Xiang

**Affiliations:** 1 Chongqing Key Laboratory of Molecular Oncology and Epigenetics, the First Affiliated Hospital of Chongqing Medical University, Chongqing, China; 2 Institute of Life Science, Chongqing Medical University, Chongqing, China; 3 Department of Oncology, the Second Affiliated Hospital of Chongqing Medical University, Chongqing, China; 4 Faculty of Health Sciences, University of Macau, Macau SAR, China

**Keywords:** CYGB, GLUT1, HXK2, p53, Breast cancer, Metabolism, Glycolysis

## Abstract

**Background:**

Recent studies suggested the globin family member cytoglobin (CYGB) as a potential tumor suppressor; however, the mechanism by which CYGB suppresses cancer is elusive. We investigated the role and mechanism of CYGB in suppressing breast cancer.

**Methods:**

CYGB expression was examined by reverse transcription PCR, quantitative reverse transcription PCR and open database analysis. Promoter methylation was examined by methylation-specific PCR. Metabolomics and proteomics were analyzed by gas chromatography-mass spectrometry and isobaric tags for relative and absolute quantitation, respectively. The effects and mechanisms of ectopic CYGB expression in breast cancer cells were assessed with molecular biological and cellular approaches in vitro and with a xenograft tumor model in nude mice*.*

**Results:**

CYGB expression was downregulated in breast cancer tissues and cell lines, which was associated with promoter methylation. Ectopic CYGB expression suppressed proliferation, migration, invasion and induced apoptosis in breast cancer cell lines MCF7 (p53WT) and MB231 (p53mt) in vitro, and inhibited xenograft tumor growth in vivo. By proteomics and metabolomics analysis, glucose metabolism was found to be one of the main pathways suppressed by CYGB. The CYGB-expressing cells had lower ATP and compromised glycolysis. Additionally, CYGB suppressed key glucose metabolism factors including GLUT1 and HXK2 in p53-dependent and -independent manners. Restoration of GLUT1 or HXK2 expression attenuated CYGB-mediated proliferation suppression and apoptosis induction.

**Conclusions:**

CYGB is a potential tumor suppressor in breast cancer that is epigenetically suppressed. The results for the first time suggest that CYGB suppresses breast cancer through inhibiting glucose metabolism, which could be exploited for breast cancer prevention and therapy.

**Electronic supplementary material:**

The online version of this article (10.1186/s13046-018-0979-9) contains supplementary material, which is available to authorized users.

## Background

Breast cancer is the most common malignancy in women across nations and races [1–3], causing tremendous physical and psychological harm and economic burden to patients and healthcare. The heterogeneous characteristics of breast cancer have severely impeded the efforts to combat this deadly disease. Despite our limited understanding of breast cancer cell biology, silencing of tumor suppressor genes (TSGs) through DNA methylation has been proven to contribute significantly to the development and progression of breast tumors [4–7].

Globins belong to a family of proteins characterized by the ability to bind and transport oxygen. Some well-known members of this family such as hemoglobin and myoglobin play crucial roles in maintaining tissue oxygenation. More recently, novel members of the family such as cytoglobin (CYGB) have been identified to play unique roles under certain conditions. CYGB expression is elevated in many cell types during tissue hypoxia and HIF1α activation [8], which is assumed to be a mechanism for protecting cells from damages caused by oxidative stresses [9, 10]. The loss of cytoglobin lead to defects and injuries organs such as heart, kidney and liver in mice [11]. Interestingly, *Cygb*^−/−^ mice developed significantly more tumors than control mice in different organs such as lung and liver, substantiating a tumor-suppressing role of CYGB in vivo [11].

Aberrant expression of CYGB has also been linked to different human cancers [12–14]. CYGB promoter region was hypermethylated in several types of solid tumors and cancer cell lines compared with normal tissue and non-tumor cell lines. However, conflicting roles for CYGB, either oncogenic or tumor suppressing, have been proposed in lung cancer [15]. The underlying mechanism by which CYGB regulates tumorigenesis has not been well elucidated. It was reported that CYGB is able to stabilize p53 after DNA damage, which promotes DNA damage repair and prevents accumulation of DNA mutation [16].

As one of the most commonly mutated genes in cancer, p53 has a broad range of impact on diverse aspects of cancer. p53 is a well-known regulator of cellular metabolism [17]. For example, many key steps of glucose metabolism such as that of aerobic glycolysis in tumor cells are simultaneously regulated by p53. Aerobic glycolysis, known as Warburg effect and a hallmark of cancer, is the main energy source of tumor cells. p53 downregulates the expression of the glucose transporter GLUT1 in tumor cells [18], thus limiting glucose intake and energy supply, and subsequently tumor growth.

To elucidate the role and underlying mechanism of CYGB in breast cancer, we examined clinical samples and human breast cancer cell lines for CYGB expression and promoter methylation, and assessed the effect of restoration of CYGB expression on proliferation in breast cancer cells. Here, we report that CYGB is epigenetically silenced and functions as a tumor suppressor to suppress breast cancer growth through inhibiting key glucose metabolizing enzymes in p53-dependent and p53-independent manners. These findings suggest CYGB functions as a tumor suppressor in breast cancer through inhibiting the glucose metabolism regulation pathway.

## Methods

### Cell lines and tissue samples

Human breast cancer cell lines (MB231, MCF7, T47D, MB468, SK-BR-3, BT549) and immortalized human mammary epithelial cell lines MCF10A and HMEC were purchased from ATCC (American Type Culture Collection). The cells were maintained in RPMI 1640 (Gibco) supplemented with 10% fetal bovine serum (FBS; Invitrogen), 100 U/mL penicillin, and 100 μg/mL streptomycin at 37 °C/5% CO_2_. Stable overexpression of CYGB and GLUT1 was generated by transfection of pcDNA3.1(+)-CYGB and pReceiver-GLUT1 plasmids into MCF7 and MB231 cells using Lipofectamine 2000 (Invitrogen). Transfected cells were then screened using neomycin and puromycin for pcDNA3.1(+)-CYGB and pReceiver-GLUT1 respectively. Empty control plasmids were used to generate control lines. The stably transfected cells were pooled and used. All tissue samples were collected at the First Affiliated Hospital of Chongqing Medical University [19].

### DNA and RNA extraction

Genomic DNA was extracted from tissue and cell samples using the DNAzol® Reagent (Invitrogen) and the QIAamp® DNA Mini Kit (Qiagen). Total RNA was isolated from tissue and cell samples using TRIzol® (Invitrogen). The concentration of RNA samples was determined by spectrophotometry using NanoDrop™ 2000 (Thermo Scientific). DNA and RNA integrity were determined using gel electrophoresis. Samples were stored at − 80 °C until utilized.

### DNA demethylation

BT549, MB231 and MCF7 cells were treated with 5-aza-2′- deoxycytidine (Aza; Sigma-Aldrich) and trichostatin A (TSA; Sigma-Aldrich) as previously described [20]. Cells were collected for DNA and RNA analyses following treatment.

### Semi-quantitative PCR and qPCR assays

Reverse transcription was performed using the Promega GoScript™ reverse transcriptase (Promega). 2 μL of cDNA (1200 ng/μL) were added to a 10 μL RT-PCR reaction mixture. *GAPDH* was used as the control. The primer sequences and specific conditions were listed in Table 1. PCR was performed using Go-Taq (Promega). Gel electrophoresis (120 V, 25 min) was performed using 2% agarose gels. Results were obtained using a BioRad Gel Doc XR+ system. Real-time PCR was performed using Maxima SYBR® Green/ROX qPCR Master Mix (Promega) according to the manufacturer’s protocol. RNA was isolated from primary breast tissues (17 paired cases of tumors and adjacent samples). The primers are listed in Table 1. Samples were amplified for 40 cycles using the 7500 Real-Time PCR System (Applied Biosystems). *β-actin* was used as the reference control.

**Table 1:** List of primers used in this study

PCR	Primer	Sequence (5′-3′)	Product size (bp)	PCR cycles	Annealing temperature (°C)
RT-PCR	CYGB-F	CTTCAGCCAGTTCAAGCACA	194	35	55
	CYGB-R	AGTACACCGGTTCCACCTTG			
	GAPDH-F	GGAGTCAACGGATTTGGT	206	23	55
	GAPDH-R	GTGATGGGATTTCCATTGAT			
qRT-PCR	HXK2-F	CCAGCTTTTGACCACATTGC	181	40	60
	HXK2-R	GTCTCTGCCTTCCACTCCACT			
	TIGAR-F	GCAGCAGCTGCTGGTATAT	241	40	60
	TIGAR-R	GTGTAAACACAGGGCACTCT			
	PGM1-F	CAGAGCAGCGCCAACTACG	222	40	60
	PGM1-R	AATGATGCAGGATACAGCAGGG			
	GLUT1-F	TCACTGTGCTCCTGGTTCTG	135	40	60
	GLUT1-R	GCTCCTCGGGTGTCTTGT			
	β-actin-F	GTCTTCCCCTCCATCGTG	113	40	60
	β-actin-R	AGGGTGAGGATGCCTCTCTT			
MSP	bCYGB-M1	GAGGTCGATCGTTAGTTCGTTC	118	40	60
	bCYGB-M2	CAACGACTAACTCGAAAACGCG			
	bCYGB-U1	GTGAGGTTGATTGTTAGTTTGTTT	120	40	58
	bCYGB-U2	CCCAACAACTAACTCAAAAACACA			

*RT-PCR* Reverse transcription-PCR, *qRT-PCR* Quantitative reverse transcription-PCR, *MSP* Methylation-specific PCR

### Methylation-specific PCR (MSP)

MSP was conducted for 40 amplification cycles using AmpliTaq®-Gold DNA polymerase (Applied Biosystems), with annealing temperatures at 60 °C and 58 °C for methylated and unmethylated samples, respectively (Table 1). Amplicons were analyzed as previously described [21].

### Cell proliferation assay

Cells were seeded in 96-well plates. After 24, 48, and 72 h, CellTiter 96® AQueous One Solution Reagent (MTS; Promega) was added according to the manufacturer’s protocol. Readings were taken after 1.5 h of incubation at 37 °C using the Infinite 200 Pro microplate reader (Tecan). Experiments were repeated three times independently.

### Colony formation assay

Stably transfected MCF7 and MB231 cells were seeded in six-well plates at 500 cells/well. Cells were cultured for 14 days, then fixed with 4% paraformaldehyde and stained with crystal violet staining. Visible colonies were counted. Assay was repeated three times independently.

### Soft agar colony formation assay

Stably transfected MCF7 and MB231 cells were plated in 6-well plates at 37 °C with 0.35% top agarose containing 1 × 10^3^ cells and 1.2% bottom agarose, with corresponding RPMI 1640 medium containing 10% fetal bovine serum in all agarose. Cell colonies were photographed after 3 weeks of incubation. Each experiment was repeated three times independently.

### Cell cycle analysis

MCF7 and MB231 cells were transfected with pcDNA3.1(+)-CYGB or pcDNA3.1(+) plasmid. 48 h post-transfection, cells were collected using 0.1% trypsin, then washed with PBS and fixed in cold 70% ethanol. Fixed cells were stained with propidium iodide (PI, 50 mg/mL) at 4 °C for 30 min and analyzed with FACS Calibur™ (BD Biosciences). Data was analyzed using the CellQuest™ software (BD Biosciences). The experiment was repeated three times independently.

### Annexin V-FITC/PI apoptosis assay

Annexin V- fluorescein isothiocyanate (FITC; BD Biosciences) and PI staining were performed according to the manufacturer’s protocol. Double-stained cells were analyzed using FACS Calibur™ (BD Biosciences). Data was analyzed using the CellQuest™ software (BD Biosciences).

### Acridine orange/ethidium bromide (AO/EB) staining

Transfected MCF7 and MB231 cells were plated in six-well plates at 1 × 10^5^ cells/well. After 24 h, the cells were washed three times with phosphate-buffered saline (PBS) and then stained with AO/EB for 5 min. Cells were visualized using a fluorescence microscope (CTR4000B, Leica). The percentages of apoptotic cells were calculated using the formula: percentage of apoptotic cells (%) = (amount of apoptotic cells/total cells examined) × 100%.

### Migration and invasion assays

Stably transfected MCF7 and MB231 cells were used for transwell cell migration and Matrigel™ invasion assays. Assays were carried out according to previously described protocol [22, 23].

### Western blot

Western blotting was done as previously described [19]. The following primary antibodies were used in this study: CYGB (Santa Cruz Biotechnology, sc-365,246), GAPDH (Cell Signaling Technology, #2118), p53 (Santa Cruz Biotechnology, sc-126), p21 (Cell Signaling Technology, #2947), β-actin (Abcam, ab8226), GLUT1 (Santa Cruz Biotechnology, sc-377,228), HXK2 (Santa Cruz Biotechnology, sc-374,091), TIGAR (Santa Cruz Biotechnology, sc-166,290).

### ATP assay

The ATP assay was performed to quantify ATP levels according to manufacturer’s instruction (#S0026, Beyotime, Shanghai, China). Briefly, 3 × 10^4^ cells were incubated with 200 μL lysis buffer reagent, then centrifuged at 12000 g × 5 min. 20 μL supernatant was mixed with 100 μL ATP working solution and incubated at room temperature for 5 min. Luminescence signal was measured with luminometer. All conditions were assayed in triplicates.

### Lactic acid assay

The level of cellular lactic acid was measured using a lactic acid assay kit (A019–2, Nanjing Jiancheng Bioengineering Institute, China). Briefly, 4 × 10^3^ cells /well were plated in a 96-well plate and cultured overnight. Then culture medium was removed and the cells were incubated in 150 μL of lysis buffer for 1 h at 37 °C. The plate was centrifuged at 400 g for 5 min, 20 μL supernatant was collected. The cell lysates were incubated with 100 μL of detection buffer at room temperature in dark. The absorbance was measured with a microplate reader at 530 nm. Lactic acid was calculated according to manufacturer’s protocol.

### In vivo tumorigenicity and IHC staining

Animal experiments were approved by the Ethics Committee of the First Affiliated Hospital of Chongqing Medical University. MB231 cells stably transfected with pcDNA3.1-CYGB or empty vector were injected subcutaneously (5 × 10^6^) into the mammary pads of 4-week-old female BALB/c nude mice [4] to generate xenograft models. Xenograft size was assessed twice a week by caliper measurement and calculated using the following equation: Volume = 0.5 × length×width^2^. The mice were sacrificed by day 20 of injection and the xenografts were removed and weighed. 4 μm-thick paraffin sections were prepared from mice #2 for immunohistochemistry (IHC) staining. IHC staining was conducted as previously described [22] using the same antibodies as Western blot.

### iTRAQ proteomics assay

Protein sample preparation, iTRAQ labeling, LC-TRIPLE TOF protein identification and bioinformatics analysis are performed as previously described [24]. Briefly, cells were lysed and sonicated for protein collection. Protein concentration was determined using the Bradford method. 8-plex iTRAQ labeling was performed following the manufacturer’s protocol (AB Scienx). Labeled peptides were fractionated, followed by reverse-phase LC-TRIPLE TOF analysis.

### GC-MS metabolomics analysis

Detailed information is presented in Additional file 1: Methods.

### Statistical analysis

Statistical analyses were performed with SPSS software for Windows, version 16 (IBM). Student’s *t*-test, the χ2 test, and Fisher’s exact test were used to evaluate assay results and compare methylation status. For all tests, a *p*-value of less than 0.05 was considered statistically significant.

## Results

### Epigenetic CYGB downregulation in breast cancer is correlated with unfavorable patient survival

**Fig. 1:**
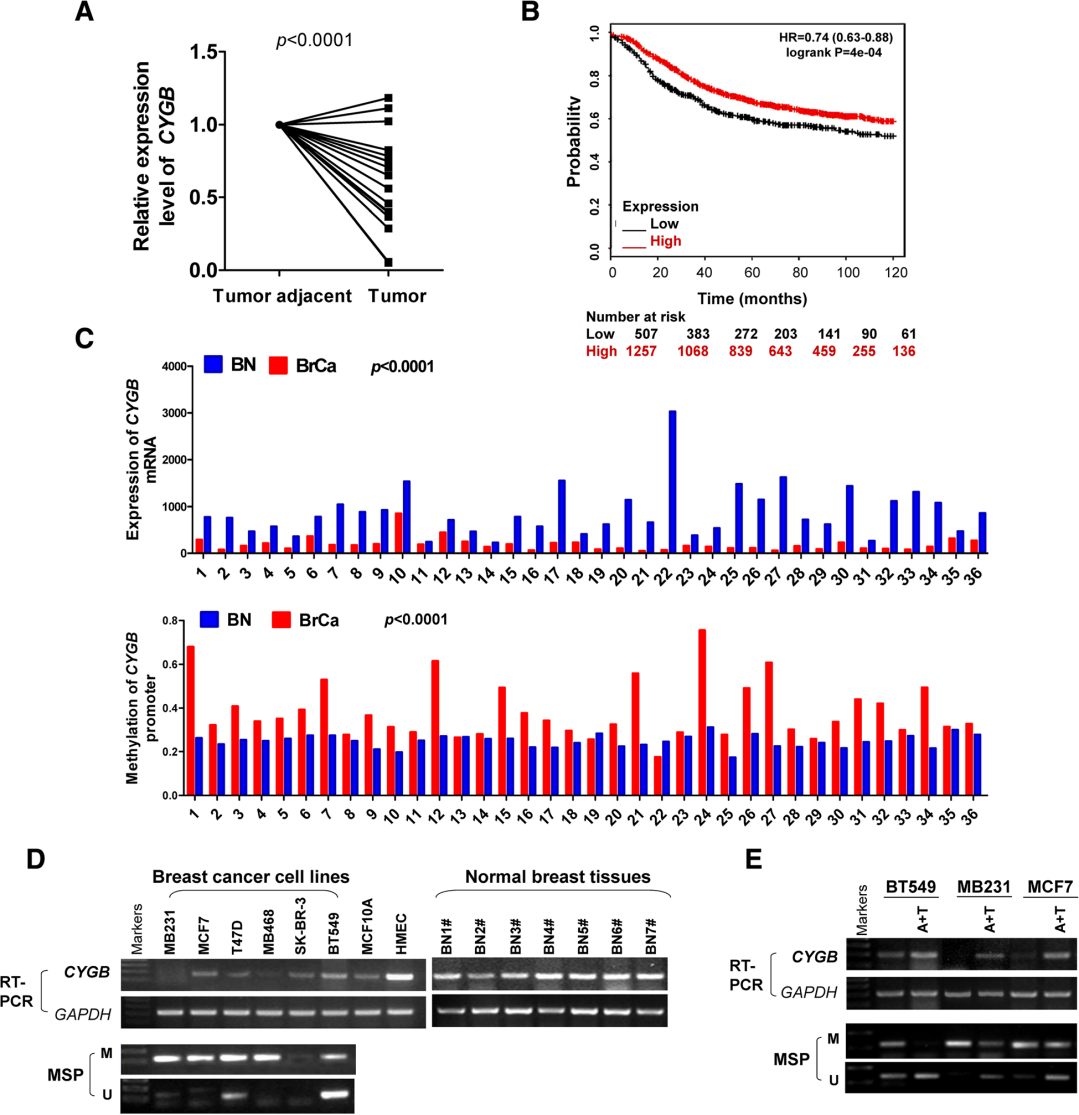
Expression and methylation of *CYGB* in breast cancer. **a** Quantitative reverse transcription-polymerase chain reaction (qRT-PCR) analysis of CYGB mRNA expression in breast tumor tissue samples and paired tumor adjacent tissue samples. **b** Low CYGB expression is correlated with poor ten-year relapse-free survival (RFS) in breast cancer patients. Prognosis data was acquired and analyzed using the Kaplan-Meier Plotter database (www.kmplot.com/analysis/index. php?p=service&cancer=breast). **c** CYGB mRNA expression and promoter methylation in paired breast cancer tissue samples and non-cancerous breast tissue samples of 36 patients from The Cancer Genome Atlas (TCGA). The data were accessed through cBioPortal (http://www.cbioportal.org/). **d** RT-PCR and MSP analyses of *CYGB* mRNA expression and promoter methylation in breast cancer cell lines. Non-tumorigenic mammary epithelial cell lines MCF10A and HMEC as well as normal breast tissue samples were used as controls. GAPDH was detected as an input control. **e** RT-PCR and MSP indicate demethylation by Aza and TSA (A + T) restored CYGB expression in BT549, MB231, MCF7 cells. GAPDH was detected as an input control. Aza: 5-aza-2′-deoxycytidine; BN: breast normal tissue; BrCa: breast cancer tissue; M: methylated; MSP: methylation-specific polymerase chain reaction; RT-PCR: reverse transcription-polymerase chain reaction; U: unmethylated

To investigate the role of CYGB in breast cancer, we first examined its expression in paired tumor and tumor adjacent tissues from 17 patients. Compared with that in breast tumor adjacent tissues, CYGB expression was significantly lower in breast tumor samples (Fig. 1a). The results were validated with The Cancer Genome Atlas (TCGA) dataset with 489 cases, which showed a significant decrease of CYGB expression in breast cancer that includes both invasive ductal breast carcinomas (IDBC) and invasive lobular breast carcinomas (ILBC) (Additional file 2: Figure S1A). Importantly, the higher CYGB expression was association with better patient survival, which was detected in the TCGA dataset with 1764 cases (Fig. 1b). A similar CYGB expression-patient survival association was also seen in an independent dataset from Kaplan-Meier Plotter with 2778 cases (data not shown). These results are consistent with literature and strongly suggest that CYGB is a potential tumor suppressor in breast cancer [13].

Previous studies have found that there are highly concentrated CpG sites susceptible to DNA methylation in the promoter region of human *CYGB*, and *CYGB* promoter methylation was found in several types of malignancies [12, 13, 25]. To investigate whether promoter hypermethylation was responsible for decreased CYGB expression in breast cancer, DNA samples from 195 breast tumors and 16 surgical margin tissue samples were subjected to MSP assay. Hypermethylation was detected in 170 (87%) breast tumor tissue samples, while only in 1 (6.7%) normal breast tissue sample (Table 2, Additional file 2: Figure S1B). Higher promoter methylation levels associated with lower CYGB expression was seen in the 36 breast tumor-normal tissue pairs from TCGA dataset (Fig. 1c). A similar trend of promoter methylation associated with reduced CYGB expression was also detected in breast cancer cell lines (Fig. 1d). In addition, CYGB expression was restored or strongly increased in BT549, MB231, and MCF7 cells by demethylation treatment with Aza and TSA (Fig. 1e). Taken together, these results suggest that CYGB is a potential tumor suppressor that is suppressed by promoter hypermethylation in breast cancer.

**Table 2:** Methylation status of the CYGB promoter in primary breast tumors

Samples	*CYGB* promoter		Frequency of methylation	*P*-value
	methylated	unmethylated		
BrCa (*n* = 195)	170	25	87%	1.56611E-18
BN (*n* = 16)	1	15	6.7%	

*BrCa* Breast cancer tissues, *BN* Breast normal tissues

### Ectopic CYGB expression suppresses tumorigenic properties of breast cancer cells through cell cycle arrest and apoptosis induction

**Fig. 2:**
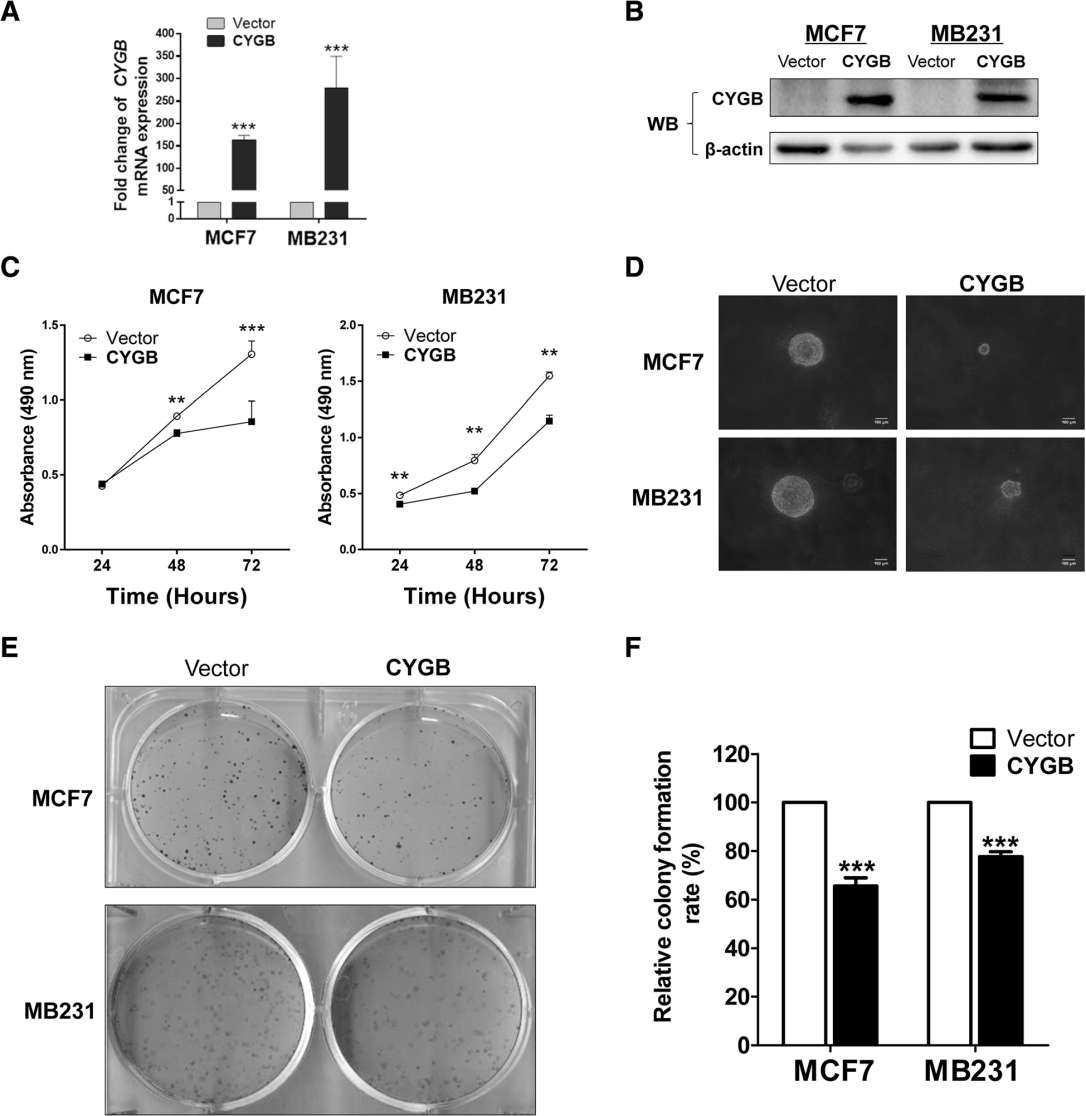
Ectopic CYGB inhibited breast cancer cell proliferation. **a** Validation of CYGB mRNA overexpression by qRT-PCR. β-actin was detected as an input control. **b** Validation of CYGB protein overexpression by Western blot. β-actin was detected as an input control. **c**, **d**, **e** and **f** Ectopic CYGB expression hindered MCF7 and MB231 growth. MCF7 and MB231 cells stably transfected with CYGB or empty vector were used for MTS assay and colony formation assay with and without soft agar to measure proliferation rates. **: *p* < 0.01 ***: *p* < 0.001. Scale bar in D indicates 100 μm

**Fig. 3:**
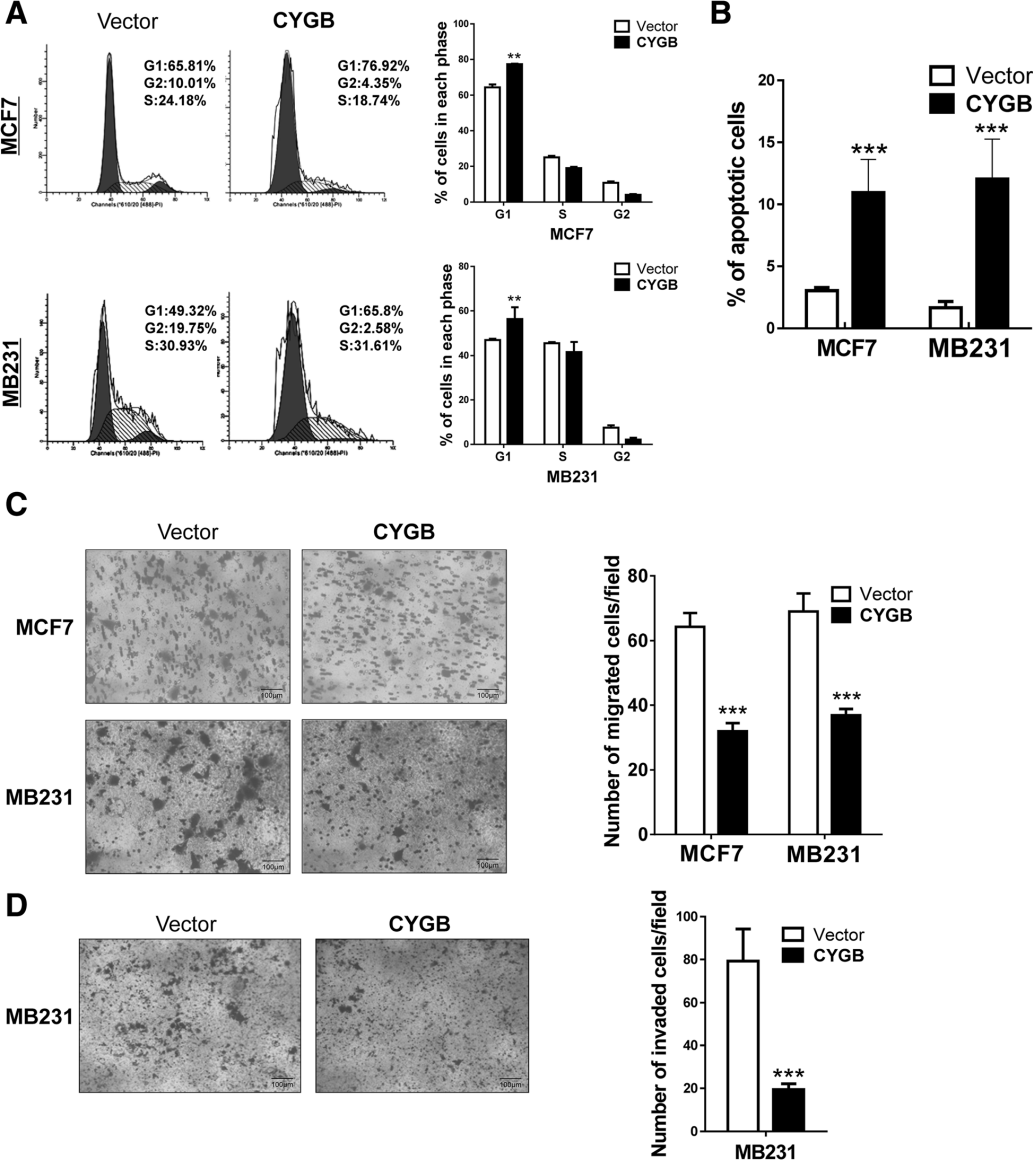
CYGB induced cell cycle arrest, apoptosis, and decreased motility in MCF7 and MB231 cells. **a** Left, distribution of cell population in various cell cycle stages. Right, quantification of cell cycle distribution. Transiently transfected MCF7 and MB231 cells were stained with propidium iodide (PI) and analyzed by flow cytometry. **b** Quantification of acridine orange/ethidium bromide (AO/EB) apoptosis staining of transiently transfected MCF7 and MB231 cells. **c** Left, representative image of transwell migration assay conducted with stably transfected MCF7 and MB231 cells. Right, quantification of migration assay by counting the number of migrated cells/field. **d** Left, representative image of transwell invasion assay conducted with stably transfected MB231 cells. Right, quantification of invasion assay by counting the number of invaded cells/field. **: *p* < 0.01 ***: *p* < 0.001

To study the cellular function of CYGB in breast cancer, MCF7 and MB231 cells were stably transfected with pcDNA3.1-CYGB or control plasmids (Fig. 2a, b) and the effect of CYGB expression restoration on cell proliferation and survival was assessed. In these cells, CYGB protein expression was increased by 25 and 7.6 fold in MCF7 and MB 231, respectively, which is comparable of the decrease fold in tumor tissues (Additional file 3: Figure S2A). Ectopic CYGB expression inhibited proliferation in both cell lines, which was detected by MTS (Fig. 2c), colony formation in soft agar (Fig. 2d), and monolayer colony formation assays (Fig. 2e, f). However, suppression of CYGB had no effect on proliferation in HMEC cells (Additional file 3: Figure S2B). Cell cycle arrest at the G0/G1 checkpoint in CYGB-overexpressing cells was detected by propidium iodide (PI) staining followed by flow cytometry assay (Fig. 3a). Higher spontaneous apoptosis rates in CYGB-overexpressing cells were detected by AO/EB staining (Fig. 3b, Additional file 4: Figure S3). Additionally, cell migration and invasion abilities were also suppressed by CYGB, which were detected by transwell assay (Fig. 3c, d). These results suggest that CYGB suppresses the malignant properties of breast cancer cells through cell cycle arrest and apoptosis induction.

### CYGB modulates pathways related to glucose metabolism in breast cancer cells

**Fig. 4:**
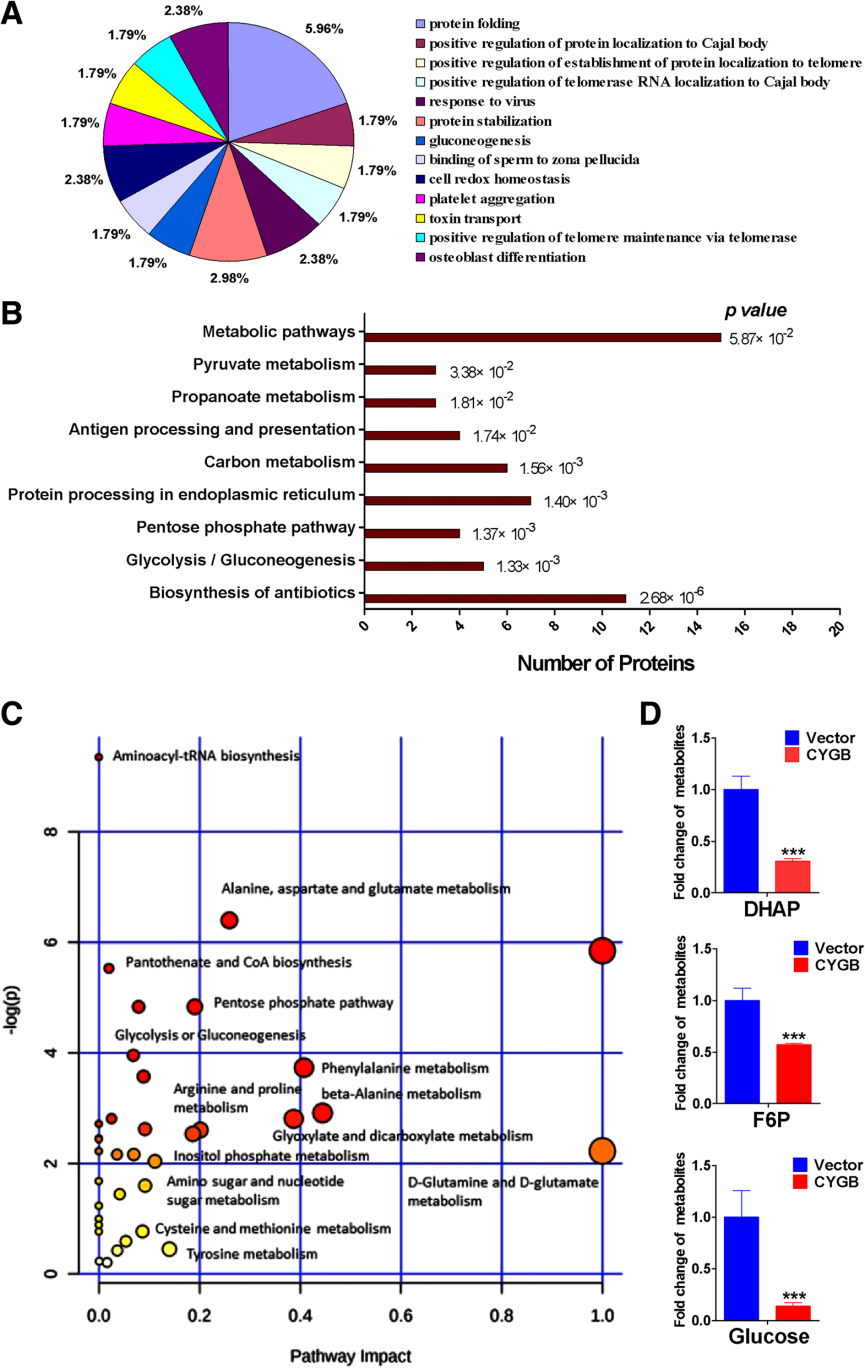
Expression proteomic and metabolomic analyses of CYGB overexpression in MCF7 cells. **a** Enrichment of differentially-expressed proteins categorized by function. **b** KEGG Pathway analysis revealed the expression of proteins involved in multiple metabolism pathways, including glycolysis/gluconeogenesis, are widely altered by CYGB. **c** and **d** Pathway analysis of differential metabolites identified by gas chromatography-mass spectrometry metabolomics assay. Data was analyzed using MetaboAnalyst software

To further explore the molecular mechanism by which CYGB suppresses breast cancer, we used the proteomics approach of 8-plex isobaric tags for relative and absolute quantitation (iTRAQ) to examine altered pathways in MCF7 cells with stable CYGB transfection. The results showed expression of 371 proteins was significantly changed after ectopic CYGB expression. The main altered pathways were related to glucose metabolism (metabolic, pyruvate, propanoate, and carbon metabolism, gluconeogenesis and glycolysis pathways, Fig. 4a, b), suggesting that CYGB plays a major role in glucose metabolism in breast cancer cells. To verify this hypothesis, GC-MS was used to detect metabolic alteration in CYGB overexpression cells. PCA, PLS-DA, and OPLS-DA evaluations showed that ectopic CYGB expression significantly increased and decreased levels of 21 and 16 metabolites, respectively. Again, the glucose metabolism pathways in CYGB-transfected cells were altered (Fig. 4c, d), which was shown as decreased intracellular glucose and its metabolites such as DHAP and fructose-6-phosphate (F-6-P; Fig. 4c, d). These results suggest that CYGB suppresses breast cancer through inhibiting glucose metabolism-related pathways.

### CYGB facilitates p53-dependent and independent regulation of glucose metabolism pathways

**Fig. 5:**
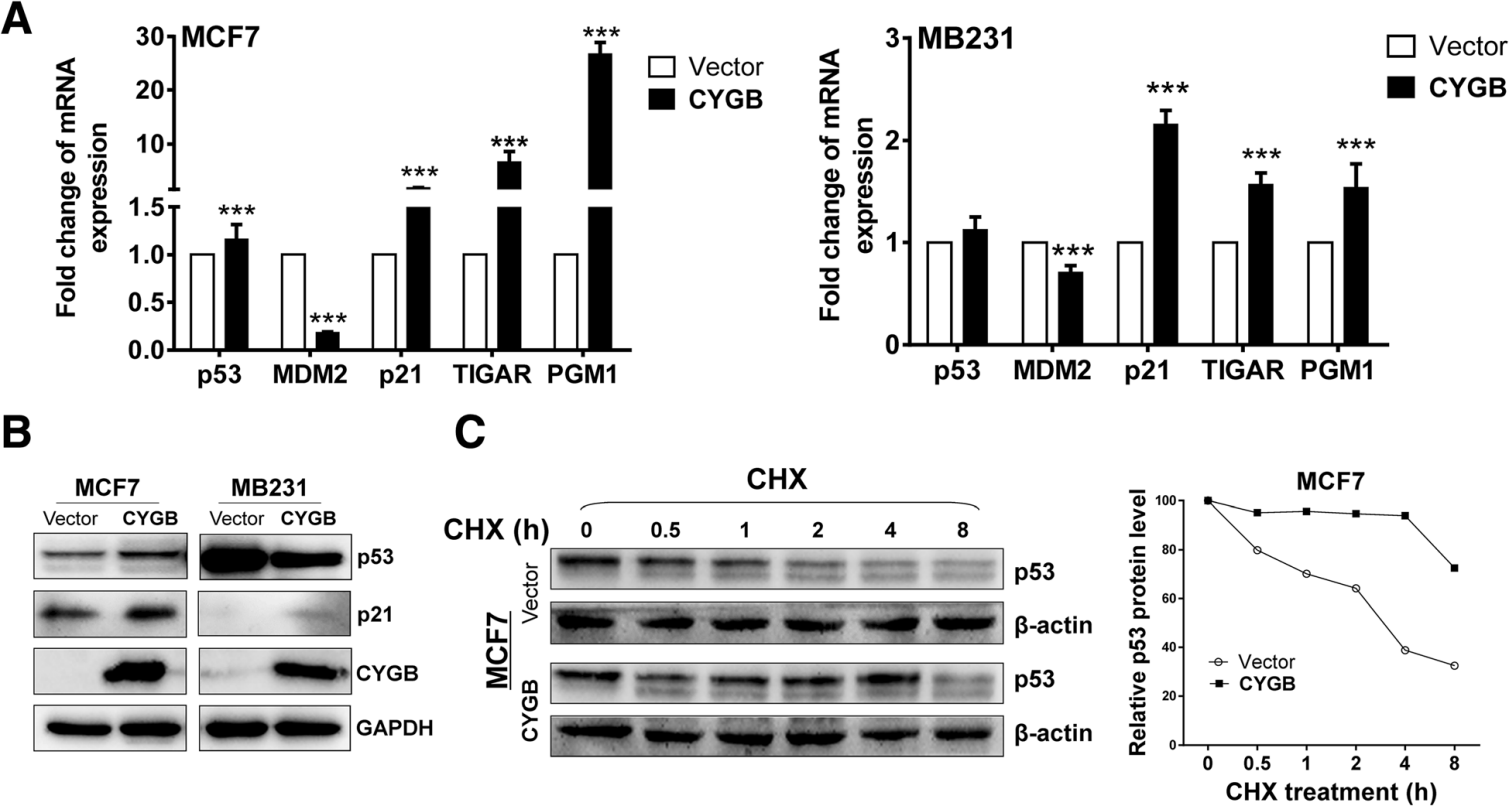
Expression of p53 and its downstream targets in CYGB-transfected MCF7 and MB231 cells. **a** mRNA expression detected by qRT-PCR. **b** Protein expression detected by Western blot. **c** p53 protein stability assay. Total protein was extracted from the cells after cycloheximide (CHX, 10 μg/mL) treatment for the indicated time periods. The indicated proteins were detected by Western blot

A previous study showed that CYGB is able to stabilize p53 in the osteosarcoma cell line U2OS [16], and p53 is well known in regulating glucose metabolism [26, 27]. Thus, we examined if CYGB modulates the glucose metabolic pathway involved p53.. Expression of p53 and downstream target p21 was used as the p53 pathway markers. Indeed, both p53 and p21 expression was elevated in ectopic CYGB expression cells. Meanwhile, the expression of the p53-inhibiting factor MDM2 was decreased (Fig. 5a). Interestingly, the expression of TIGAR and PGM1, glucose metabolism regulators downstream of p53, was increased by 10 and 26 folds, respectively (Fig. 5a), suggesting that p53 plays a role in CYGB-mediated glucose metabolism regulation. p53 protein was stabilized by CYGB, consistent with decreased MDM2 in MCF7 cells (Fig. 5c). Altogether, these results suggest CYGB suppresses glucose metabolism at least in part through p53 in breast cancer cells have wild-type (WT) p53. However, it was noted that expression of p21, TIGAR and PGM1 was also slightly increased by 1.5–2 folds in MB231 cells that have the loss-of-function p53 R280K mutation (Fig. 5b), implying that CYGB may also regulate glucose metabolism through an alternative p53-independent mechanism.

### CYGB inhibits glucose metabolism through downregulating GLUT1 and HXK2

**Fig. 6:**
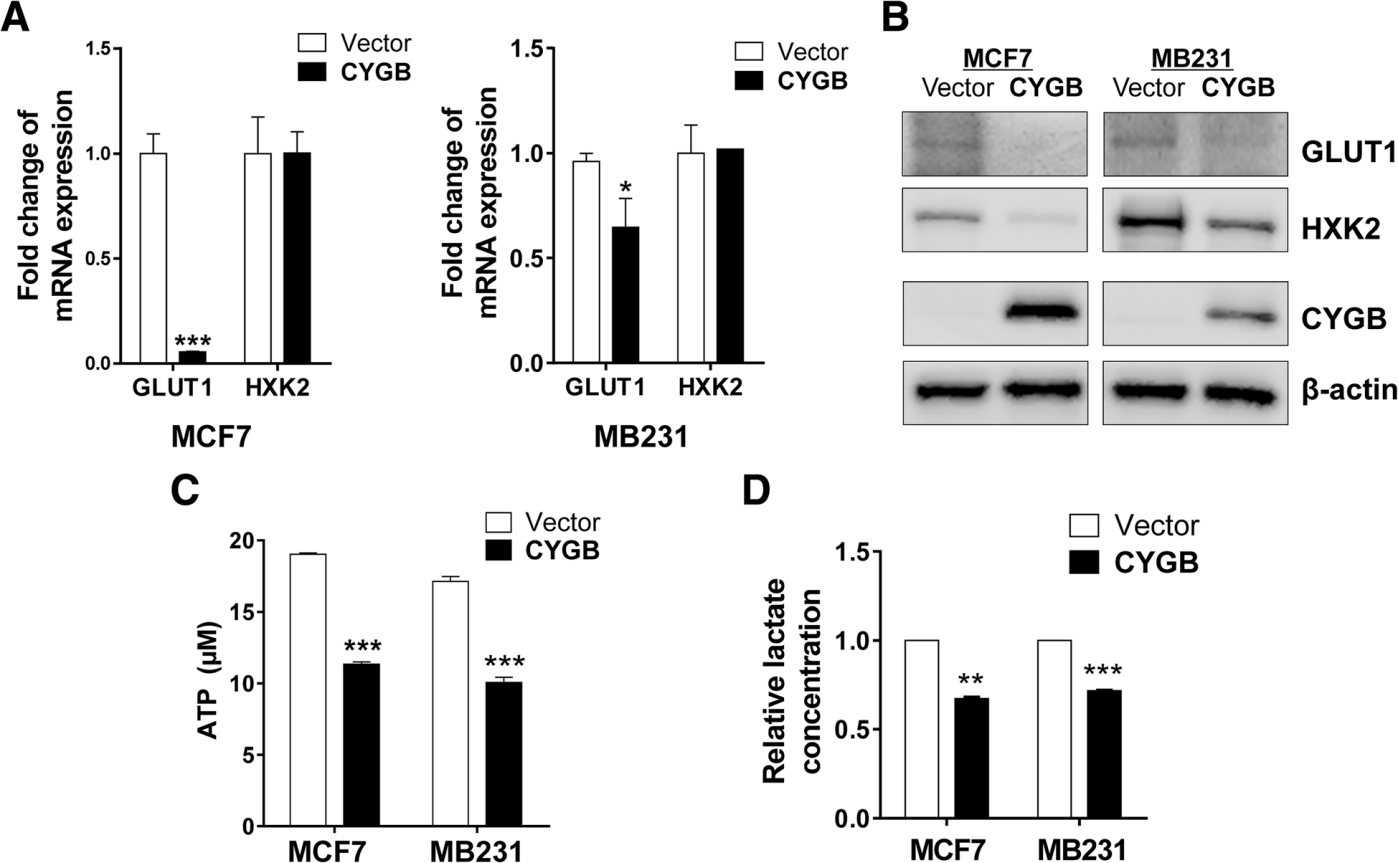
Reduced glucose metabolism in CYGB-transfected cells. **a** mRNA expression was detected by qRT-PCR. **b** Protein expression was detected by Western blot. **c** Level of ATP in transfected MCF7 and MB231 cells. **d** Relative lactate concentration was calculated by taking the value of the control vector transfected cells as 1. *: *p* < 0.05; **: *p* < 0.01; ***: *p* < 0.001

Based on that intracellular glucose was reduced and glycolysis was suppressed by CYGB restoration, we further investigated key factors in glucose metabolism, focusing on GLUT1 (the main glucose transporter) and HXK2 (key enzyme in the first step of glucose metabolism). GLUT1 mRNA and protein was significantly decreased by CYGB in MCF7 and MB231 cells (Fig. 6a, b). There was a decrease in HXK2 at the protein level while the mRNA level remained unaffected (Fig. 6a, b). Functional assays and gene expression analysis showed similar relationship of p53 and GLUT1 or HXK2: an inverse association of expression between CYGB and either GLUT1 or HXK2 was found in the TCGA breast cancer dataset (Accessed through www.cbioportal.org, Additional file 5: Figure S4). This notion was supported by that GLUT1 and HXK2 expression was reduced in CYGB-transfected p53-null cells (Additional file 6: Figure S5).

The inhibition of GLUT1 expression by CYGB was well correlated to reduced cellular glucose (Fig. 4d). The cellular ATP level was reduced in ectopic CYGB expression cells (Fig. 6c), consistent with suppressed glucose metabolism. Lactate was reduced, reflecting reduction of glycolysis in ectopic CYGB-expressing cells (Fig. 6d). Taken together, these results suggest CYGB suppresses GLUT1-mediated glucose intake and metabolism pathways mediated by HXK2.

### Ectopic GLUT1 and HXK2 expression partially reverses the tumor suppressing function of CYGB

**Fig. 7:**
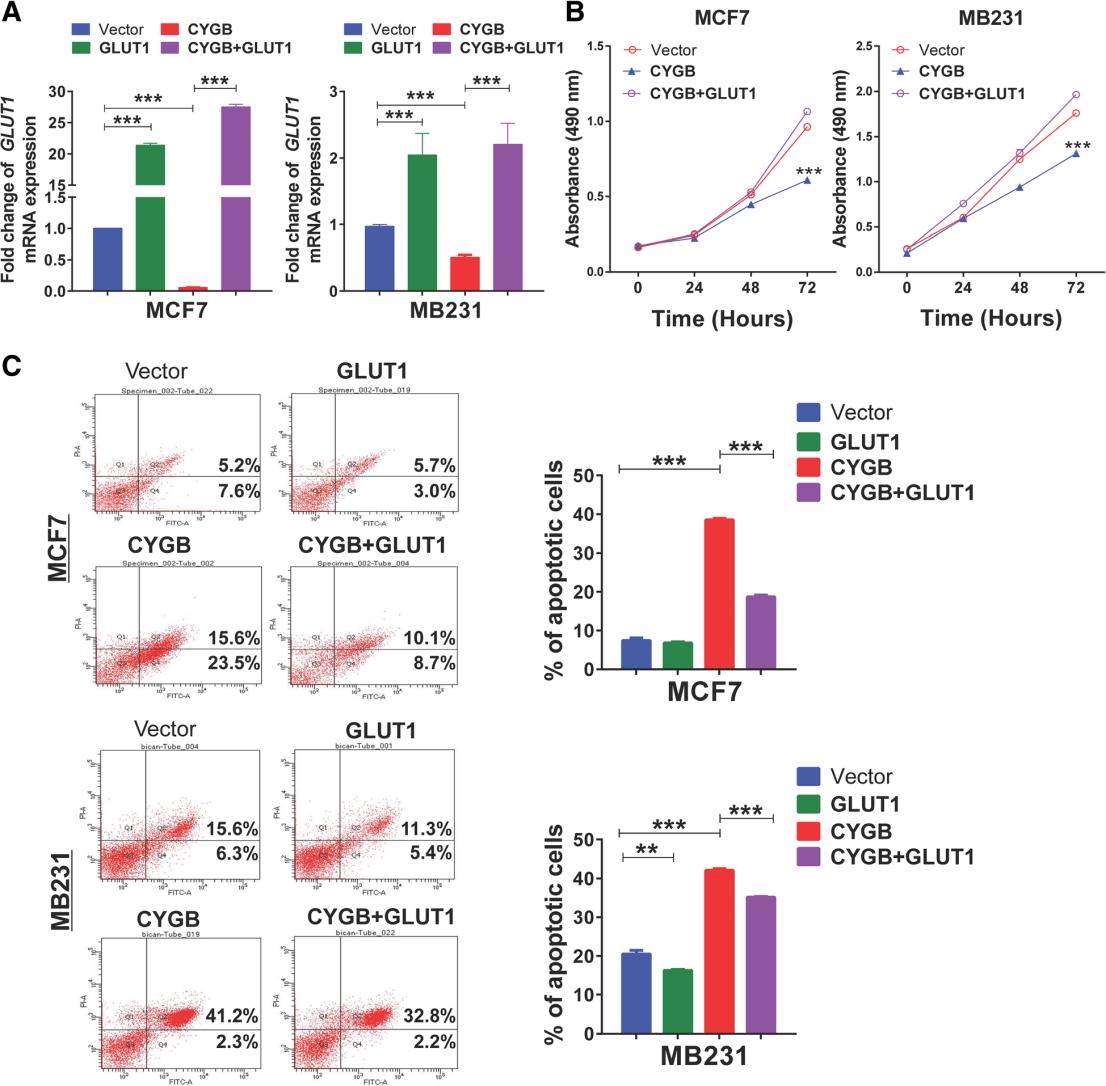
Ectopic GLUT1 expression partially reversed CYGB’s effect on proliferation and apoptosis. **a** Verification of GLUT1 overexpression by qRT-PCR. **b** Effect of GLUT1 overexpression on proliferation in MCF7 and MB231 cells. **c** Effect of GLUT1 overexpression on apoptosis in MCF7 and MB231 cells. **: *p* < 0.01; ***: *p* < 0.001

We further validated the role of GLUT1 and HXK2 in CYGB-mediated tumor suppression. Ectopic expression of GLUT1 promoted proliferation while suppressed apoptosis in CYGB-overexpressing cells (Fig. 7a, b, c). Similar rescue effect was observed with HXK2 overexpression (Additional file 7: Figure S6A, B, C). These results support that CYGB inhibits breast tumor cell growth through suppression of glucose metabolism involving GLUT1 and HXK2.

### CYGB suppresses xenograft breast tumor growth in nude mice

**Fig. 8:**
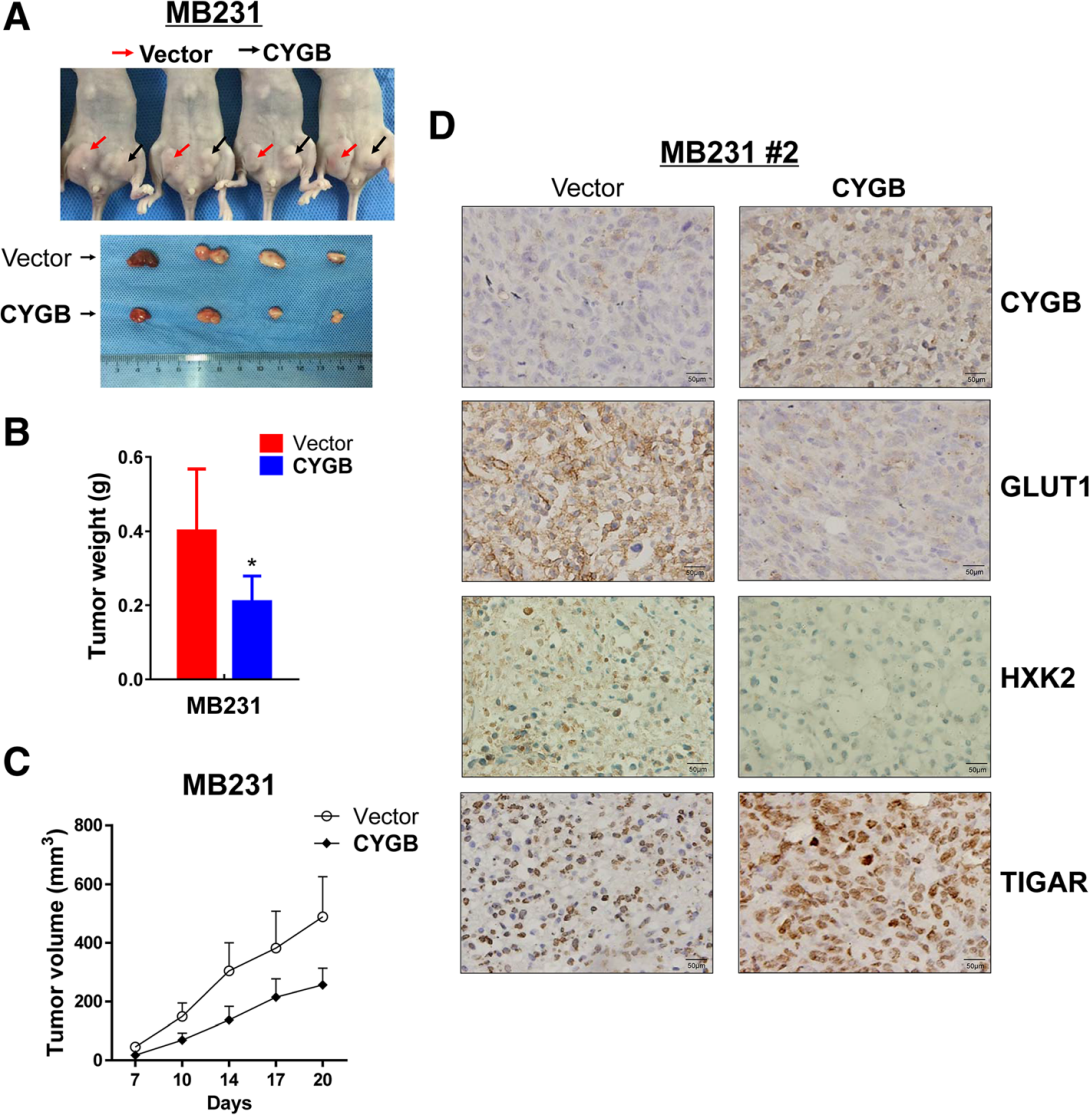
CYGB suppressed xenograft tumor growth in vivo. **a** Image of xenografts before and after resection. Red and black arrows indicate empty vector control tumors and CYGB-overexpressing tumors, respectively. **b** Tumor weight (4 xenografts/group). **c** Growth curve of xenograft tumors. **d** Representative images of immunohistochemistry (IHC) staining. Paraffin sections were prepared from the tumors of mice #2 and stained for CYGB, GLUT1, HXK2, and TIGAR. *: *p* < 0.05

**Fig. 9:**
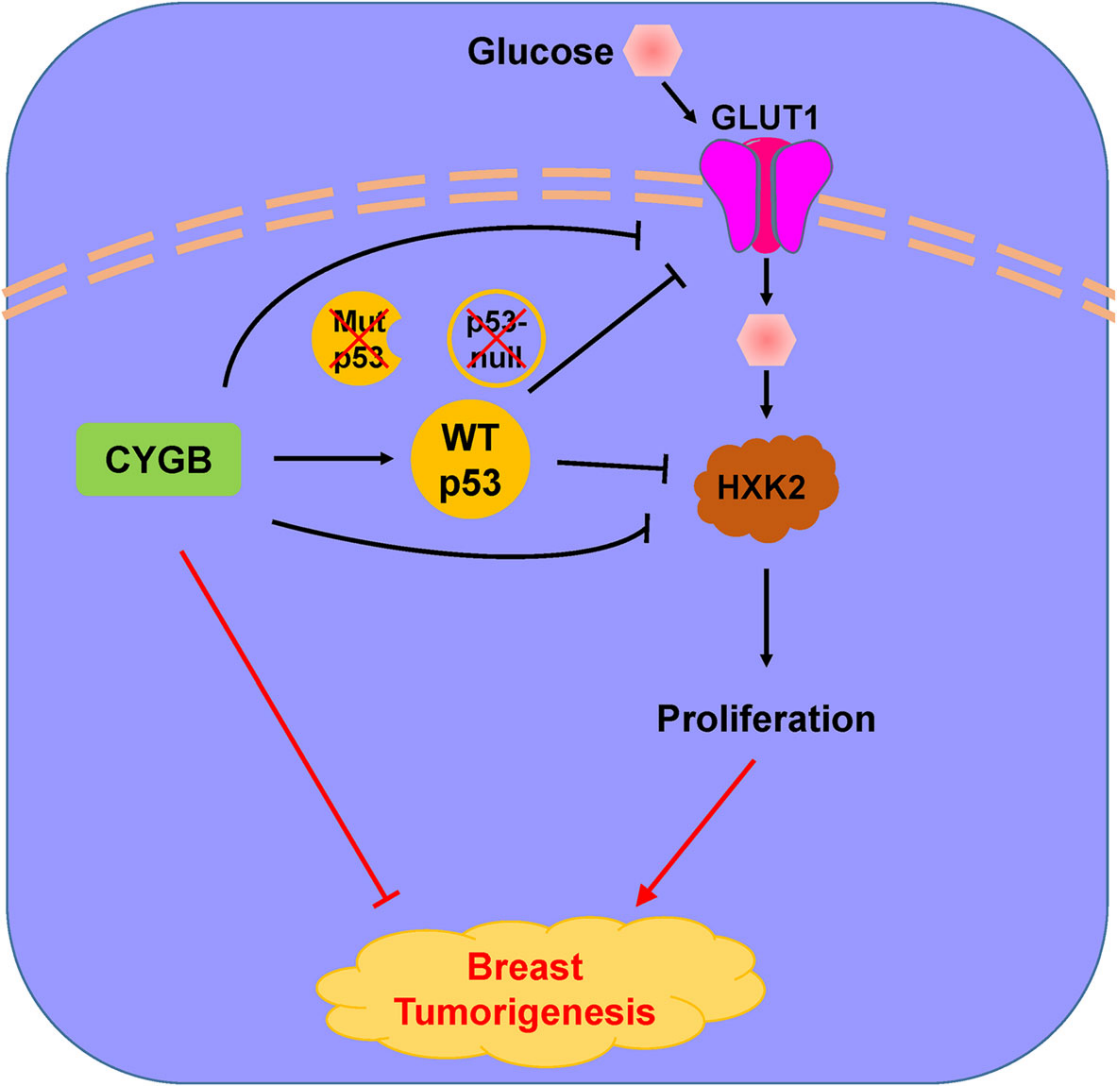
Schematic illustration of the role of CYGB in breast cancer as indicated by this study

To explore the functions of CYGB in vivo, the nude mice xenograft model was used. Tumors derived from CYGB-expressing cells grew slower, which was shown as smaller tumor volume and weight than that of tumors derived from the control vector-transfected cells (Fig. 8a, b, c). IHC staining of the CYGB-expressing tumors showed lower GLUT1 and HXK2 and higher TIGAR expression (Fig. 8d), substantiating that CYGB suppresses breast cancer through the regulation of these key glucose metabolism factors. Figure 9 summarized the conclusions indicated by in vitro and in vivo results in this study.

## Discussion

In this study, we provide evidence supporting CYGB as a tumor suppressor in breast cancer: CYGB expression was downregulated in breast cancer tissues and cell lines, which was associated with promoter methylation. Ectopic CYGB expression suppressed the malignant properties of breast cancer cells both in vitro and in vivo. Additional studies for the first time suggest that CYGB suppresses breast cancer through inhibition of glucose intake and metabolism involving GLUT1and HXK2 in p53-dependent and -independent manners. Our results shed lights on the mechanism of breast cancer development and suggest that the CYGB-mediated regulation of glucose metabolism could be a target for breast cancer prevention and therapy.

It is not uncommon that many promoter hypermethylated genes in tumors function as tumor suppressors, and restored expression of these genes may suppress tumor initiation and/or progression [6, 20, 21, 28, 29]. Consistent with literature that CYGB is epigenetically downregulated in several types of malignancies [12, 13, 25, 30–32], we detected CYGB down-regulation associated with promoter hypermethylation in breast cancer cell lines and tissues. Demethylation treatment effectively restored CYGB expression, confirming that promoter methylation contributes to suppression of CYGB expression in breast cancer cells. CYGB suppressed the malignant properties in breast cancer cells both in vitro and in vivo. Thus, our results suggest that *CYGB* is a potential epigenetically suppressed tumor suppressor gene in breast cancer.

As a member of the hexacoordinate globin superfamily, CYGB was shown to protect cells against hypoxic stress [15, 33], and inhibit the PI3K/AKT/mTOR pathway [34]. However, how CYGB suppresses tumor growth remains elusive. Thus, we took proteomic and metabolomic approaches to explore how CYGB executes its cellular functions. Both screening results pointed to genes and pathways related to glucose metabolism. Further results showed that CYGB-expressing cells have lower glucose and ATP and compromised glycolysis, confirming that CYGB suppresses glucose metabolism. As a major energy and biogenesis material source for most cellular functions and proliferation, glucose metabolism is key to malignant cells that have high proliferation rates. Specifically, we found CYGB suppressed the expression of glucose transporter GLUT1 and the key glycolysis enzyme HXK2. Therefore, we focused on glucose metabolism to elucidate the tumor suppressing mechanism of CYGB, and identified p53-dependent and -independent pathways downstream of CYGB.

The tumor suppressor p53 modulates multiple cellular functions and plays a major role in glucose metabolism [35, 36]. Consistent with previous studies indicating CYGB positively regulates p53 in both human and mouse cells [14, 16], our results show that CYGB activated WT p53 in MCF7 cells, and in turn, p53 regulated the expression of metabolism regulating factors GLUT1, HXK2, TIGAR, and PGM1 [18], The mechanism for CYGB-mediated p53 activation remains to be fully determined, although we found both p53 mRNA and protein are increased by ectopic CYGB expression in MCF7 cells. Consistent with increasing p53-mediated p21 expression, CYGB induced cell cycle arrest at the G1/S checkpoint. It is noteworthy that p21 and other metabolism regulating factors such as GLUT1 and HXK2 were also regulated by CYGB in p53-mutated MB231 cells, suggesting that CYGB suppresses metabolism through a p53-independent mechanism in p53-mutated breast cancer cells, which deserves further studies.

We further confirmed the involvement of the glucose metabolism pathway in CYGB’s tumor suppression by restoration of GLUT1 and HXK2 expression. The relatively limited extent of effect in reversing apoptosis and cytostasis by expression of each of these factors individually could be due to that this pathway is cooperatively regulated by many regulators. It should be noted that, although we focus on glucose metabolism in the current study, other mechanisms are not excluded. Further studies are warranted to determine if CYGB-mediated glucose metabolism regulation pathway can be targeted for breast cancer prevention and therapy.

## Conclusions

Our results confirmed that promoter hypermethylation contributes to CYGB suppression in breast cancer and shed lights on the mechanism by which CYGB inhibits breast cancer growth. CYGB plays an important role in metabolic reprogramming, through regulating glucose metabolism in breast cancer with or without the involvement of p53. It would be interesting to determine if the CYGB-mediated glucose metabolism regulation pathway can be a valuable potential target for breast cancer prevention and therapy.

## Additional files

Additional file 1: Detailed description of the GC-MS metabolomics assay method. (DOC 36 kb)

Additional file 2: **Figure S1.** *CYGB* expression and promoter methylation in breast cancer. (A) Analysis of CYGB mRNA expression in normal breast and IDBC/ILBC tissue samples from TCGA. Data accessed through the Oncomine database (www.oncomine.org). (B) Representative images of MSP for detecting *CYGB* promoter methylation in 195 breast tumor tissue samples and 16 surgical margin tissue samples. BN: breast normal tissue; IDBC: invasive ductal breast carcinoma; ILBC: invasive lobular breast carcinoma. (TIF 4125 kb)

Additional file 3: **Figure S2.** (A) Semi-quantification of CYGB overexpression Western blot results. CYGB expression in vector-transfected cells were set as 1. (B) Suppression of CYGB in HMEC does not affect cell proliferation. Representative images of AO/EB staining of MCF7 and MB231 cells transfected with CYGB or control plasmid. The cells were cultured on coverslips, stained with AO/EB and photographed under a fluorescence microscope. (TIF 1224 kb)

Additional file 4: **Figure S3.** Representative images of AO/EB staining of MCF7 and MB231 cells transfected with CYGB or control plasmid. The cells were cultured on coverslips, stained with AO/EB and photographed under a fluorescence microscope. (TIF 1275 kb)

Additional file 5: **Figure S4.** Inverse association between CYGB and (A) GLUT1 and (B) HXK2 expression in breast cancer. TCGA breast cancer data set accessed through cBioPortal (www.cbioportal.org) was analyzed. (TIF 814 kb)

Additional file 6: **Figure S5.** Overexpression of CYGB in p53-null H1299 cells suppressed GLUT1 and HXK2 expression in p53-null H1299 cells. GLUT1 expression in CYGB/H1299 cells was too low for detection. (TIF 571 kb)

Additional file 7: **Figure S6.** Ectopic HXK2 expression partially reversed CYGB’s effect on proliferation and apoptosis. (A) Verification of HXK2 overexpression by qRT-PCR. (B) Effect of HXK2 overexpression on proliferation in MCF7 and MB231 cells. (C) Effect of HXK2 overexpression on apoptosis in MCF7 and MB231 cells. *: *p* < 0.05; **: *p* < 0.01; ***: *p* < 0.001. (TIF 9380 kb)

## Abbreviations

AO/EB: Acridine orange/ethidium bromide
Aza: 5-aza-2′-deoxycytidine
CHX: Cycloheximide
CYGB: Cytoglobin
GC-MS: Gas chromatography–mass spectrometry
IDBC: Invasive ductal breast cancer
ILBC: Invasive lobular breast cancer
iTRAQ: Isobaric tags for relative and absolute quantitation
MSP: Methylation-specific polymerase chain reaction
OPLS-DA: Orthogonal projections to latent structures discriminant analysis
PCA: Principal component analysis
PLS-DA: Partial least squares discriminant analysis
qRT-PCR: Quantitative reverse transcription polymerase chain reaction
RFS: Relapse-free survival
RT-PCR: Reverse transcription polymerase chain reaction
TCGA: The cancer genome atlas
TSA: Trichostatin A
