# Supplementary material for: The epigenetically downregulated factor CYGB suppresses breast cancer through inhibition of glucose metabolism
Source: J Exp Clin Cancer Res. 2018 Dec 13;37:313. doi: 10.1186/s13046-018-0979-9 (PMC6293581; doi:10.1186/s13046-018-0979-9)
Supplement: Supplementary file 1 — Detailed description of the GC-MS metabolomics assay method. (DOC 36 kb) [file 13046_2018_979_MOESM1_ESM.doc]

**Supplementary Methods**

**GC-MS sample preparation**

Frozen cell mixture was ultrasonicated for 2 min, and then placed at -40 °C for 2 min. This procedure was repeated 7 times. The mixture was placed at 4 °C prior to centrifugation at 16,000g and 4 °C for 15 min. Then, 200 µL of the combined supernatant was added to a GC vial containing 10 µL of internal standards (0.05 mg/mL of 13C6-L-leucine and 13C6-15N-L-isoleucine). The mixture was dried under gentle nitrogen stream. The dry residue was mixed with 30 µL of 20 mg/mL methoxyamine hydrochloride in pyridine, and the resulting mixture was vortexed vigorously for 30s and incubated at 37 °C for 90 min. A 30 µL of N,O-Bis (trimethylsilyl) trifluoroacetamide (BSTFA; with 1% Chlorotrimethylsilane) was added into the mixture and derivatized at 70 °C for 60 min prior to performing GC-MS metabolomics analysis. Quality control sample pooled from representative supernatants of cell samples in each group was prepared and analyzed with the same procedure as the experiment samples.

**GC-MS analysis**

Metabolomics instrumental analysis was performed on an Agilent 7890A gas chromatography system coupled to an Agilent 5975C inert MSD system (Agilent Technologies Inc., CA). A HP-5ms fused-silica capillary column (30 m × 0.25 mm × 0.25 µm; Agilent J&W Scientific, Folsom, CA) was utilized to separate the derivatives. Helium (>99.999%) was used as a carrier gas at a constant flow rate of 1 mL/min through the column. Injection volume was 1 µL in split l mode with a ratio of 2:1, and the solvent delay time was 6 min. The initial oven temperature was held at 70 °C for 2 min, ramped to 160 °C at a rate of 6 °C/min, to 240 °C at a rate of 10 °C/min, to 300 °C at a rate of 20 °C/min, and finally held at 300 °C for 6 min. The temperatures of injector, transfer line, and electron impaction source were set to 250 °C, 260 °C, and 230 °C, respectively. The impact energy was 70 eV, and data was collected in a full scan mode (m/z 50-600).

**Data pre-processing of GC-MS**

The typical total ion current (TIC) chromatograms were illustrated in Figure 1. The peak picking, alignment, deconvolution, and further processing of raw GC-MS data were referred to the previous published protocols (1). The final data was exported as a peak table file, including observations, variables, and peak abundances. The data were normalized against total peak abundances before performing univariate and multivariate statistics.

**Statistical analysis and identification of differential metabolites**

For multivariate statistical analysis, the normalized data were imported to SIMCA software (version 13.0, Umetrics, Umeå, Sweden), where the data were preprocessed by unit variance scaling and mean centering before performing PCA, PLS-DA, and OPLS-DA. The model quality is described by the R^2^X or R^2^Y and Q2 values. R^2^X (PCA) or R^2^Y (PLS-DA and OPLS-DA) is defined as the proportion of variance in the data explained by the models and indicates the goodness of fit. Q^2^ is defined as the proportion of variance in the data predictable by the model and indicates the predictability of current model, calculated by cross-validation procedure. In order to avoid model over-fitting, a default 7-round cross-validation in SIMCA software was performed throughout to determine the optimal number of principal components. The variables with variable importance in the projection values larger than 1 and p values of univariate statistical analysis lower than 0.05 based on the OPLS-DA model were identified as potential differential metabolites. Fold change was calculated as binary logarithm of average normalized peak intensity ratio between the two groups, where the positive value means that the average mass response of vector group is higher than CYGB group.

**Structural identification of metabolites**

The structural identification of differential metabolites was performed as follows. The AMDIS software was applied to deconvolute mass spectra from raw GC-MS data, and the purified mass spectra were automatically matched with an in-house standard library including retention time and mass spectra, Golm Metabolome Database, and Agilent Fiehn GC/MS Metabolomics RTL Library.

1. Gao X, Pujos-Guillot E, Sebedio JL. Development of a quantitative metabolomic approach to study clinical human fecal water metabolome based on trimethylsilylation derivatization and GC/MS analysis. Anal Chem. 2010;82(15):6447-56.
