# Supplementary figures and images for: The epigenetically downregulated factor CYGB suppresses breast cancer through inhibition of glucose metabolism
Source: J Exp Clin Cancer Res. 2018 Dec 13;37:313. doi: 10.1186/s13046-018-0979-9 (PMC6293581; doi:10.1186/s13046-018-0979-9)

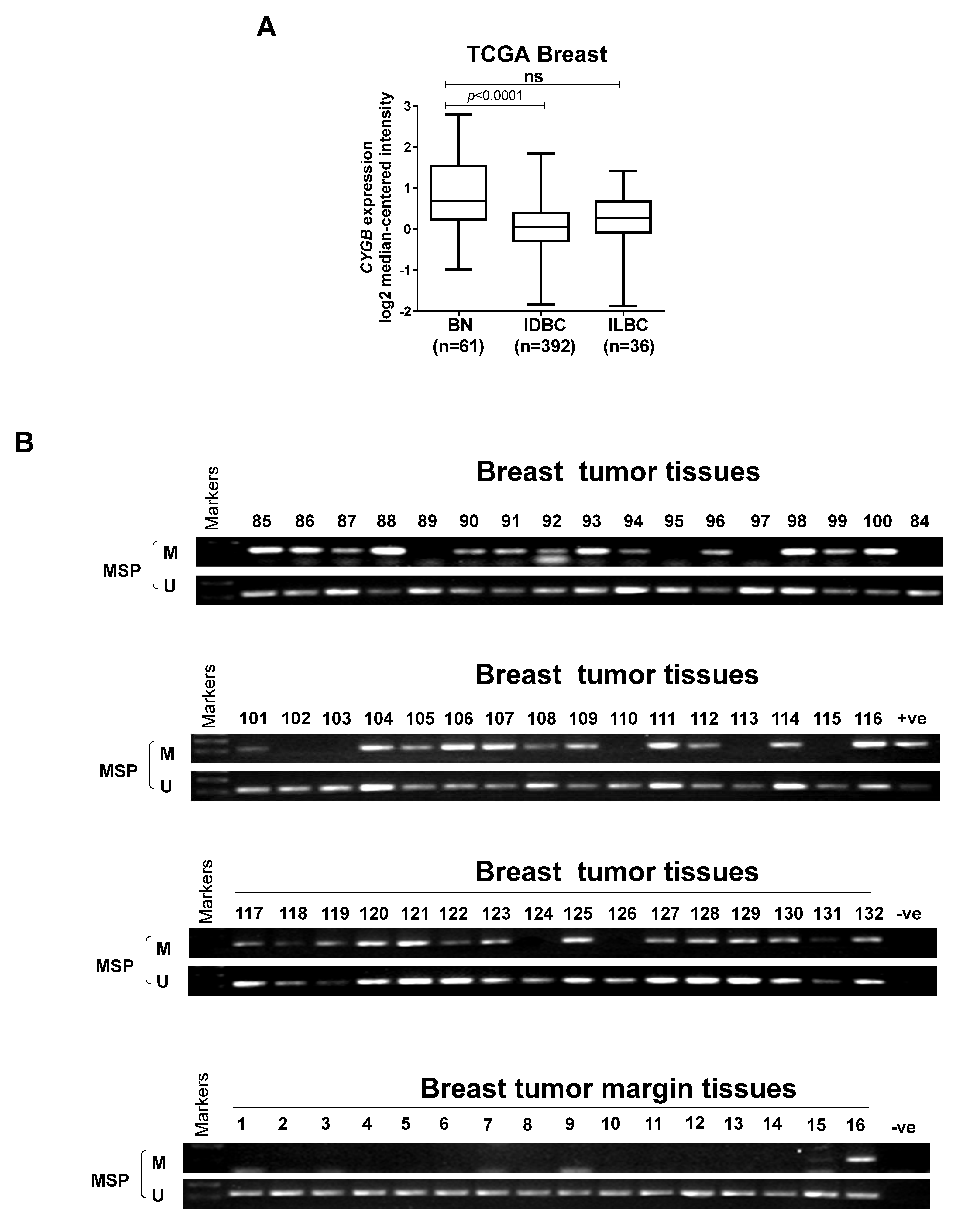

Supplement: Supplementary file 2 — Figure S1. CYGB expression and promoter methylation in breast cancer. (A) Analysis of CYGB mRNA expression in normal breast and IDBC/ILBC tissue samples from TCGA. Data accessed through the Oncomine database (www.oncomine.org). (B) Representative images of MSP for detecting CYGB promoter methylation in 195 breast tumor tissue samples and 16 surgical margin tissue samples. BN: breast normal tissue; IDBC: invasive ductal breast carcinoma; ILBC: invasive lobular breast carcinoma. (TIF 4125 kb) [file 13046_2018_979_MOESM2_ESM.tif]

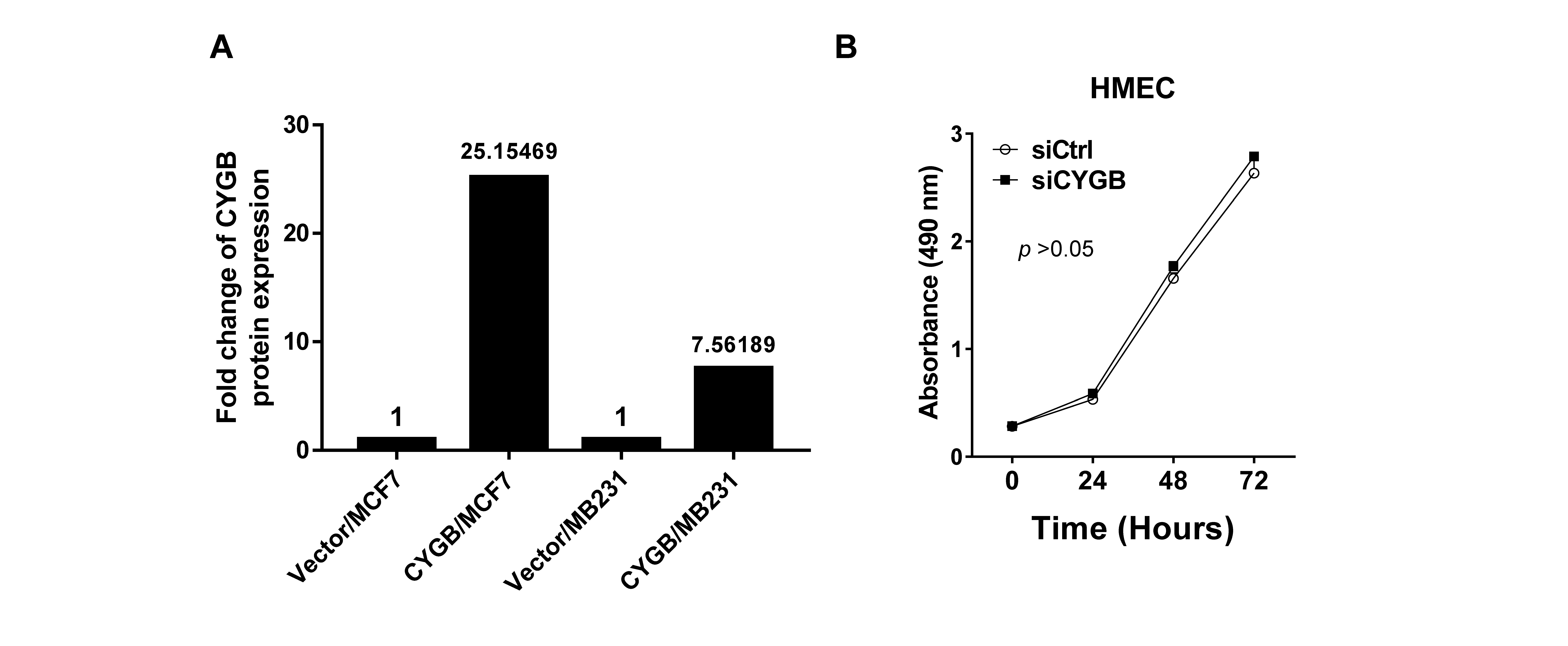

Supplement: Supplementary file 3 — Figure S2. (A) Semi-quantification of CYGB overexpression Western blot results. CYGB expression in vector-transfected cells were set as 1. (B) Suppression of CYGB in HMEC does not affect cell proliferation. Representative images of AO/EB staining of MCF7 and MB231 cells transfected with CYGB or control plasmid. The cells were cultured on coverslips, stained with AO/EB and photographed under a fluorescence microscope. (TIF 1224 kb) [file 13046_2018_979_MOESM3_ESM.tif]

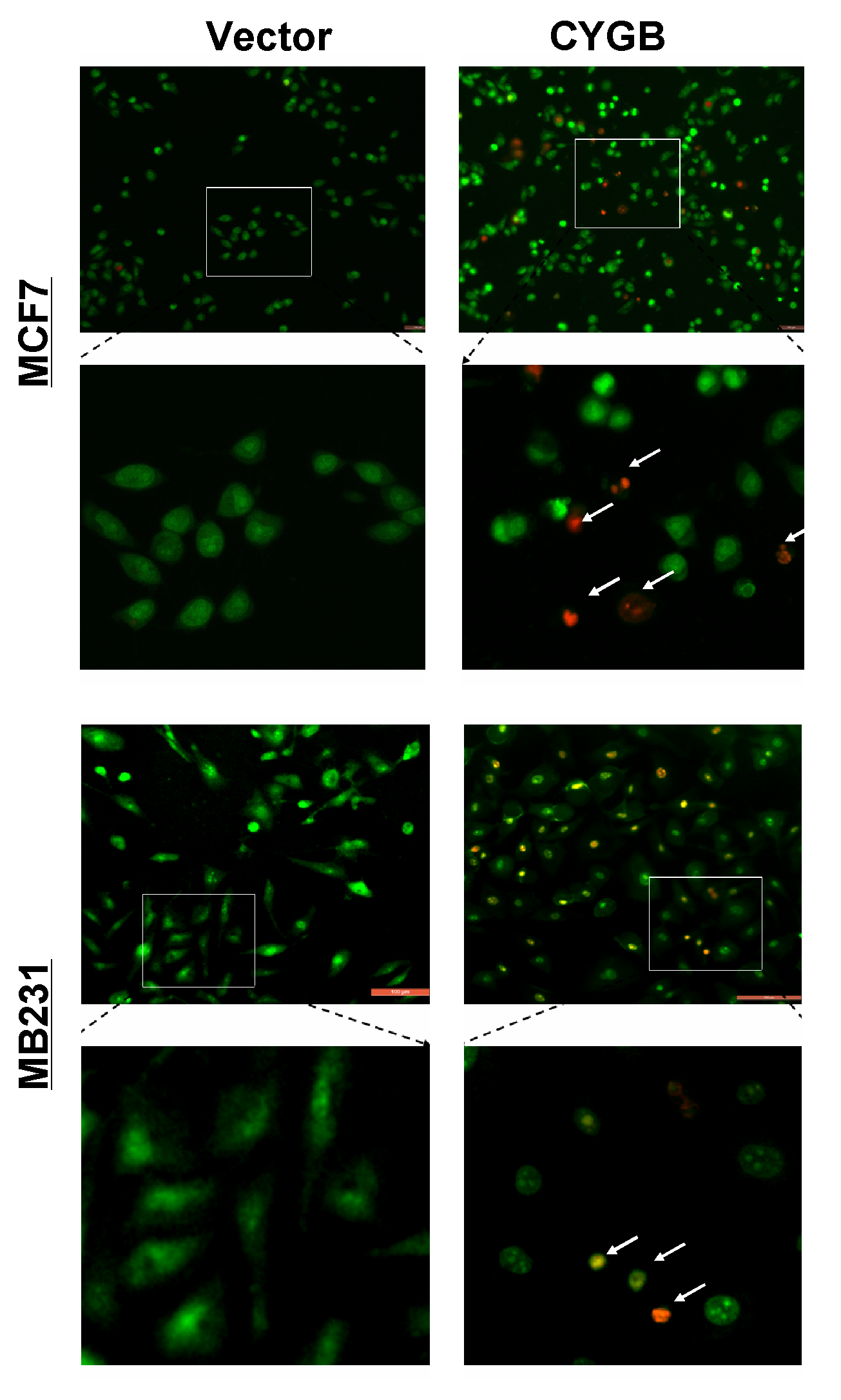

Supplement: Supplementary file 4 — Figure S3. Representative images of AO/EB staining of MCF7 and MB231 cells transfected with CYGB or control plasmid. The cells were cultured on coverslips, stained with AO/EB and photographed under a fluorescence microscope. (TIF 1275 kb) [file 13046_2018_979_MOESM4_ESM.tif]

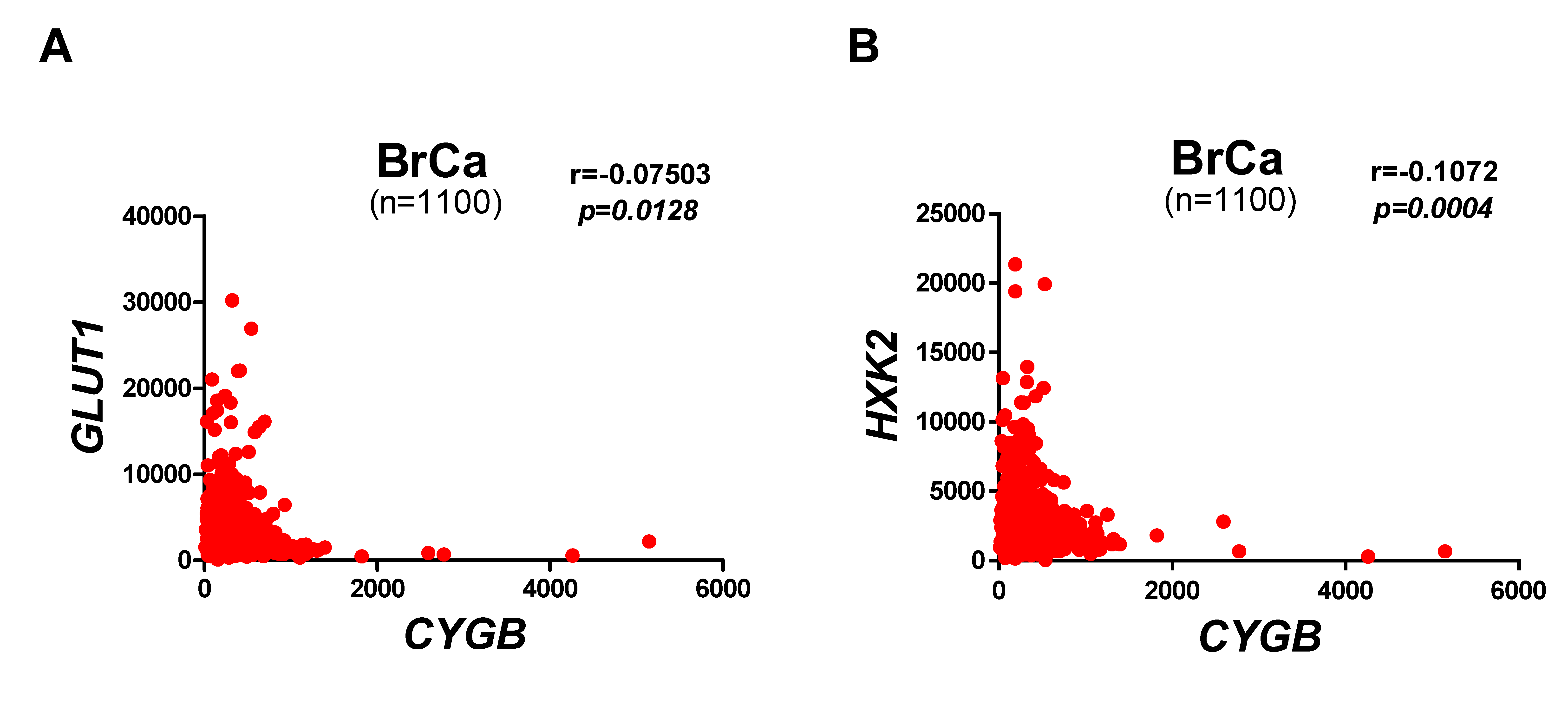

Supplement: Supplementary file 5 — Figure S4. Inverse association between CYGB and (A) GLUT1 and (B) HXK2 expression in breast cancer. TCGA breast cancer data set accessed through cBioPortal (www.cbioportal.org) was analyzed. (TIF 814 kb) [file 13046_2018_979_MOESM5_ESM.tif]

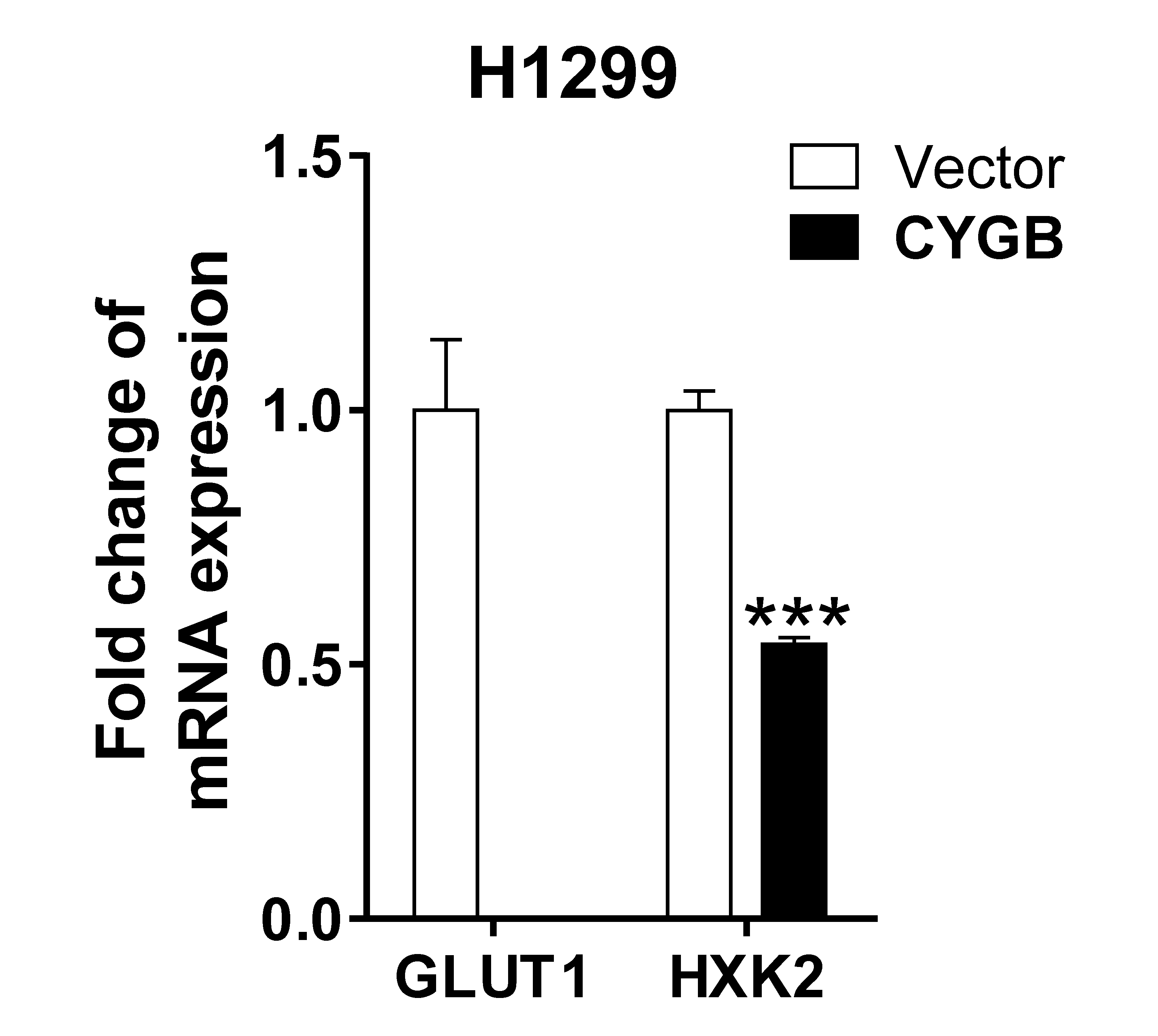

Supplement: Supplementary file 6 — Figure S5. Overexpression of CYGB in p53-null H1299 cells suppressed GLUT1 and HXK2 expression in p53-null H1299 cells. GLUT1 expression in CYGB/H1299 cells was too low for detection. (TIF 571 kb) [file 13046_2018_979_MOESM6_ESM.tif]
